# Automated Translation of Chronic Disease Diagnosis Codes using the ChatGPT Large Language Model

**DOI:** 10.23889/ijpds.v9i5.2759

**Published:** 2024-09-18

**Authors:** Zoe Hsu, Cassandra Chisholm, Conné Lategan, Eddy Lang, Xiaoming Wang, Erik Youngson

**Affiliations:** 1 The Alberta Strategy for Patient Oriented Research Support Unit; 2 Provincial Research Data Services, Alberta Health Services; 3 Faculty of Medicine, University of Alberta; 4 Department of Emergency Medicine, University of Calgary

## Background

The International Classification of Diseases (ICD) is revised over time and there are region-specific versions, including ICD-10-CA (Canada) and ICD-9-CM (USA). Studies spanning multiple ICD versions require crosswalks to translate diagnosis codes across versions, but manual crosswalk development is costly and requires clinical expertise.

## Objective

To evaluate the accuracy of a pre-trained large language model (LLM) to automatically translate chronic disease diagnosis codes from ICD-10-CA to ICD-9-CM.

## Approach

Eight prompts were developed to instruct the OpenAI Generative Pre-trained Transformer 4 (GPT-4) LLM to translate 1,272 ICD-10-CA codes for the Elixhauser Comorbidity Index to ICD-9-CM. Prompt accuracy (%) was measured against a crosswalk developed by the Canadian Institute of Health Information. Variability was assessed by replicating each prompt three times. Mean accuracy ± standard deviation was reported for each prompt across replications, for both five-digit and truncated three-digit codes.

## Results

The highest prompt performance was observed when assigning a persona of a medical coding specialist (40.8% ± 0.9%), requesting justification for the selected code (41.4% ± 1.1%), and providing diagnosis code labels (47.5% ± 0.7%). For truncated three-digit codes, these prompts achieved accuracy of 82.0% ± 0.5%, 80.8% ± 0.9%, and 82.7% ± 0.1%, respectively. Combining these three prompting techniques marginally improved accuracy to 48.6% ± 0.7% for five-digit codes and 84.3% ± 0.2% for truncated three-digit codes.

## Conclusion

General-purpose LLMs are currently not sufficiently accurate at automating ICD code translation for chronic diseases.

### Implications

Additional experiments with fine-tuning, task-specific training, and prompt engineering are needed to improve accuracy and reduce variability.

